# Research and the Medical Student

**Published:** 1921-08-27

**Authors:** 


### RESEARCH AND THE MEDICAL STUDENT.

Sufficient time has now elapsed since what one may term the restarting of the pre-war medical schools to provide a fairly clear idea of the future of medical research and its relation to the student. We write this note, therefore, essentially for the guidance of the parent, himself a layman, who sees in medicine a possible career for his son. For, in the popular acclamation of all recent suggestions concerning research work, there lies a danger both of misunderstanding the real meaning of research and the difficulties to be overcome before any professional man can advantageously take part in it. Also there is some confusion between the relative values in service to mankind of investigational work carried on in an organised institution and the careful, painstaking practice of medicine among the general public. At the outset let us emphatically state that the latter is not an inferior branch of the profession, and it is in no sense wise to allow the younger generation of doctors to attempt, as some undoubtedly do, to avoid it at all costs.

But to confine ourselves strictly to the prospects in the purely investigational field, let us consider in the first place their bearing upon the new student himself. To start with, his training up to two or three years is really an introduction to the methods of thought on scientific subjects in general. Admittedly, those matters chosen are the most likely to benefit his after-work, but they are essentially preparatory. When sitting for his earliest examinations it is more a rapid adaptability to the methods of the scientific world than a power to repeat in detail a number of accepted facts, which will mark the beginner as likely, one day, to achieve investigational distinction. It is, indeed, surprising how few of the men at the head of those early examination lists ultimately provide the world with a successful contribution in medical research. And in any case it is certainly their teachers alone who can at this stage make even an approximately reliable forecast of the student's future.

The next stage of training, which is carried out while in actual contact with the sick, is decidedly more helpful. Through the whole of the time in the wards as clerk, dresser, or newly qualified house officer the ultimate moulding of a research worker occurs. At the end of that time the constitution of our great hospitals is such that, should a man prove himself specially adapted for some special work, other circumstances allowing it, he is likely to be invited to join one of the many "teams," or already established investigational units, at work in medicine's multiple problems. It may be he is placed permanently on the "staff" of this hospital, or his invitation may be of a more temporary nature; but in any case the principle is the same. The new-comer must prove himself capable, and then receive the invitation of his colleagues to join them in their work.

One makes this, to the professional man, apparently redundant point, because the popular idea of the brilliant and solitary worker is so remarkably persistent and yet in practice almost never seen. However brilliant an examinee a boy may have been, the odds are all against his beginning, or for that matter continuing, as an individualist. The intricacies of modern medical problems rarely allow of this method, and as an instance one may mention the organisation of the Medical Research Council, the new units working at the voluntary hospitals, and the manifold results of "team-work" investigations during the war.

Lastly, the parent must remember that only the most suitable men will be chosen for these research appointments. One does not mean that they are the "best" men in the general sense, but that they must be the best for the particular type of work. Such suitability can be judged only by their professional colleagues, and a vague "liking for research" can in no sense be placed in the balance as a favourable indication for committing a schoolboy to a medical career.

The next part in connection with research—one true to-day, at least—is that, crudely put, there is no money in it. Gradually matters are improving, in the sense that Government grants, wise distributions of legacies, and other controlled sources of income are being devoted to payment of men proved efficient at their work. The expenses—and they are often enormous—are being met in a variety of ways. But in any case the research worker, if he is genuinely such, can rarely expect more than the "living wage" when compared with his professional brethren of similar mental calibre. The whole system of professional work, the fact that the endeavour is medical and towards the general welfare of mankind, practically precludes the personal development and exploitation of any discoveries made. No one cavils at this, no one wishes it to be otherwise, but herein lies a great difference from research in the majority of other "non-human" sciences.

It is a recognised fact that successful scientific research and financial worries are, speaking generally, incompatible. The undivided interest in the work for its own sake, the careful consideration of generalities, and painstaking inspection of the minutest experimental details can never occur except in circumstances of unhampered concentration. The difficulty of procuring such conditions is one of the greatest disadvantages under which medical research in this country now labours. So many suggested revisions have been put forward, Sir Ronald Ross and others have so ably urged the workers' case, that the public knows it in outline well enough. Will the parent of the academically "clever" boy also realise this weakness when the glamour of research attracts the youthful fancy? If private means are his inheritance as well as the power to think connectedly, there is little more to be said; the widest investigational fields may be open to him, and his life may be well and happily devoted to them. But to the great average, the majority who have all their own way to make and themselves entirely to maintain, we would urge that the magic of medical research be forgotten either as an attraction to entering the profession or as an easy path to fame for the "clever" boy. If medicine, the healing and helping of our fellow-men, attracts, let us open-mindedly join its ranks, for, meanwhile, we can rest assured that if circumstance and our professional ability allow, sooner or later the chance for research will come. Then is the time for decision. In our student days we can but work, perfect our training, and leave our preceptors to judge.

